# Supplementary material for: A set of multi-entry identification keys to African frugivorous flies (Diptera, Tephritidae)
Source: Zookeys. 2014 Jul 24;(428):97–108. doi: 10.3897/zookeys.428.7366 (PMC4143993; doi:10.3897/zookeys.428.7366)
Supplement: Supplementary material 4 — Key to Capparimyia [file zookeys-428-097-s004.zip › SF4_ZooKeys_key to Capparimyia/lucid_player/help/help/about_features_and_entities.htm]

About Entities and Features


# About Entities and Features

*Entities* in Lucid are the things
the key aims to identify. Depending on the scope of the key, the entities
may be different kinds of things. For example, in a key to the palms of
Brazil, the entities will be species of palms, in a key to the families
of flies of the world the entities will be fly families, in a key to minerals
the entities will be types of minerals, in a key to disorders of sweet
potatoes the entities will be the causes of disorders found in sweet potatoes.
In Lucid3 we use the term *entity*
because it applies to all the possible things that a key may be designed
to help identify. Lucid3 itself doesn't know whether the entities are
species of plants, families of flies, minerals or diseases � they are
all simply entities to Lucid3, and are all handled in the same way.

*Features* in Lucid3 are characteristics
of the entities in the key that can be used to describe each entity.

For example, in a key to minerals one feature may be the types of crystalline
form shown by the mineral. Amongst the minerals in the key, there will
be a number of different crystal forms � cubic, tetragonal etc. The different
possibilities for crystal form are called states of the feature. States
of a feature are indicated in the Lucid Player by scoring boxes preceding
the state names.

In Lucid3 there are three main types of features.

*Multi-state features*, like
those shown for the crystal form of minerals, have two or more alternative
states.

*Numeric features*, by contrast,
are ones in which real numbers (integer or decimal) are used to describe
the entities.  Numeric
features are normally used for lengths, widths, ratios and counts. For
example, a key to butterflies may use a numeric feature for the length
of the forewing, or for the number of segments in the antennae.

*Grouping features* are features
that contain other features.
